# Prolonged wireless measurement of intragastric pH and pharmacodynamics of extended-release injectable omeprazole in horses

**DOI:** 10.1093/jvimsj/aalag287

**Published:** 2026-11-12

**Authors:** Evelyn Hodgson, Marthe Thirouin, Tallia-Rume Romano, Allison Stewart, Stephanie Bond, Jessica Wise

**Affiliations:** School of Veterinary Science, Faculty of Science, University of Queensland, Gatton, Queensland 4343, Australia; School of Veterinary Science, Faculty of Science, University of Queensland, Gatton, Queensland 4343, Australia; School of Veterinary Science, Faculty of Science, University of Queensland, Gatton, Queensland 4343, Australia; School of Veterinary Science, Faculty of Science, University of Queensland, Gatton, Queensland 4343, Australia; School of Veterinary Science, Faculty of Science, University of Queensland, Gatton, Queensland 4343, Australia; School of Veterinary Science, Faculty of Science, University of Queensland, Gatton, Queensland 4343, Australia

**Keywords:** EGGD, EGUS, equine, ESGD, gastric, ulcers

## Abstract

**Background:**

Adaptation of wireless capsules for intragastric pH measurement in horses has a short, variable attachment duration. Prolonged measurement is important for pharmacodynamic studies. Extended-release injectable omeprazole (ERIO) induces acid suppression for ≤ 7 days. Five-day dosing intervals are now recommended for improved healing rates.

**Hypothesis/Objectives:**

To (1) optimize the methodology to achieve 5-7 days of capsule attachment; (2) determine whether capsules can detect a treatment effect of ERIO; and (3) describe the pharmacodynamics of ERIO.

**Animals:**

Twelve healthy adult horses.

**Methods:**

Capsules were attached transendoscopically to the glandular mucosa on day 0 using either 1 hemostasis clip (1HEM; *n* = 4), 4 hemostasis clips (4HEM; *n* = 5), or a helix tacking device (TACK; *n* = 9). Intragastric pH was continuously recorded and ERIO was administered on day 1. Data are reported as median (IQR).

**Results:**

Capsules remained attached and obtained data for 51 h (42-65), 129 h (105-256), and 95 h (71-154) for 1HEM, 4HEM, and TACK, respectively. The percentage of time the pH was above 4 (%tpH > 4) was significantly higher (*P* < .001) on day 1 (82.0% [72.7-91.2]) than day 0 (14.3% [7.1-23.5]). Complete recordings were available for 6/12 horses throughout the 5-day dosing interval; %tpH > 4 exceeded 66% for 5 days after ERIO in 4/6 horses (66.7%).

**Conclusions and clinical importance:**

These refined, minimally invasive attachment techniques enabled longer, more consistent intragastric pH measurement. A treatment effect associated with ERIO administration was detected and %tpH > 4 exceeded 66% throughout the 5-day dosing interval in most horses.

## Introduction

Equine gastric ulcer syndrome (EGUS) is a highly prevalent condition in horses.^1^ The pathogenesis is associated with gastric acidity, particularly with squamous disease,^2^ and acid suppression is the cornerstone of treatment regardless of lesion type.^1^ Extended-release injectable omeprazole (ERIO) is used as an alternative to PO formulations, especially in refractory cases.^3^ This drug induces acid suppression for up to 7 days, as shown in a preliminary study using percutaneous endoscopic gastrostomy (PEG) tubes to measure intragastric pH.^4^ Superior EGUS healing rates have been reported with 5- rather than 7-day dosing intervals of ERIO, but this study did not include pharmacodynamic data.^5^ A target threshold for healing is often extrapolated from human medicine as the percentage of time the pH is above 4 (%tpH > 4) exceeding 66%.^6^ Intragastric pH measurement is therefore crucial to our understanding of EGUS and its treatment. Nasogastric pH probes are commonly used but the electrode location is unknown, and the probes are prone to displacement and breakage.^7–10^ Percutaneous endoscopic gastrostomy tubes^11^^,^^12^ and gastric cannulas^13–16^ allow a fixed electrode location but are invasive. A wireless capsule, designed for intraesophageal pH measurement in humans,^17^ recently was adapted for use in horses by attaching the capsule to the gastric mucosa using hemostasis clips.^18^ This minimally invasive method provided a known electrode location, but attachment duration was short and variable with only 53% of capsules achieving ≥ 24 h.^18^

Although wireless capsules show promise as a noninvasive alternative to existing methods of measurement, longer attachment duration is needed. Longer recording durations are particularly important for interventional and pharmacodynamic studies. Larger hemostasis clips designed to incorporate more tissue to improve retention achieved attachment durations of 5-7 days in foals.^19^ New devices are emerging on the human market that are outperforming hemostasis clips; one example is a transendoscopic helix tacking device that deploys barbed helical tacks into the tissue tethered with suture.^20–22^

Our objectives were to: (1) use and compare the larger hemostasis clips and helix tacking device to optimize the methodology and achieve 5-7 days of capsule attachment; (2) determine whether the capsules could detect a treatment effect of ERIO; and (3) describe the effect of ERIO on intragastric pH. We hypothesized that the tacking device could achieve a longer attachment duration than the clips, that the capsules could detect a treatment effect, and that ERIO would induce acid suppression for at least 5 days in all horses.

## Materials and methods

The study design is illustrated in Figure 1, and a detailed description of the methodology is included in the Appendix.

**Figure 1:**
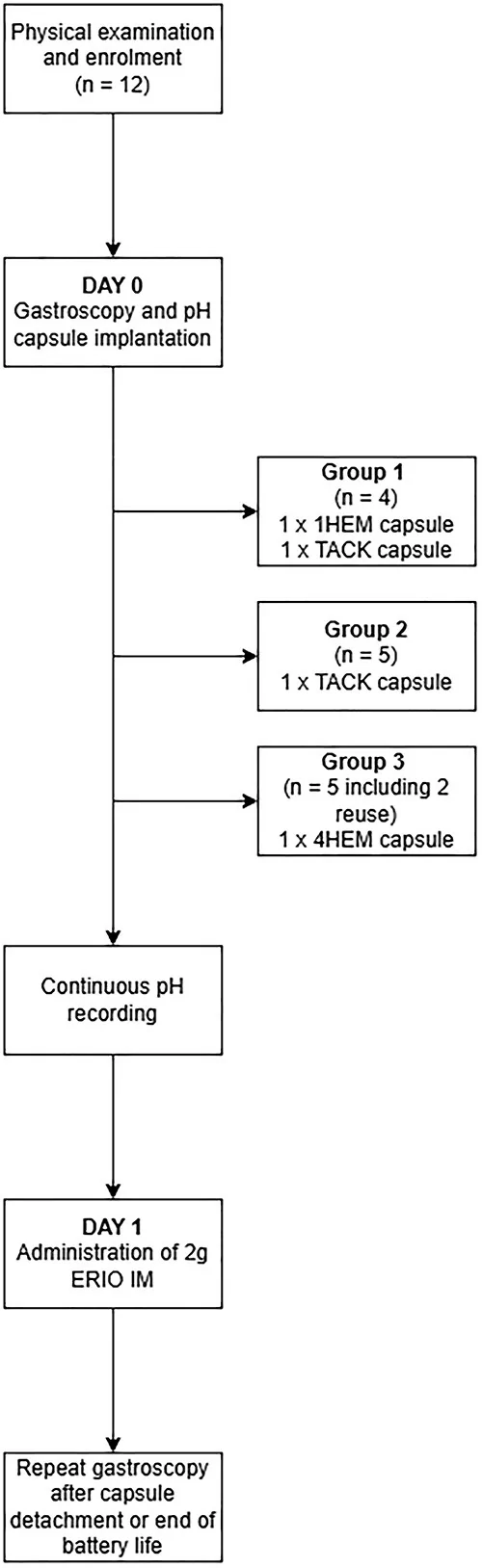
Flowchart showing the study design including capsule attachment methods according to group, ERIO treatment, pH recording, and monitoring. Abbreviation: ERIO = extended-release injectable omeprazole.

### Study cohort and design

Twelve healthy adult mares (8 Standardbreds and 4 Australian Stock Horses) from the University of Queensland Equine Unit, aged 6-27 years and weighing 400-525 kg, were enrolled in a prospective interventional study. Horses were housed in groups of 2-4 in grass paddocks with ad libitum lucerne hay and free access to fresh water. The University of Queensland Animal Ethics Committee approved the research protocol (2024/AE000419).

For direct method comparison, 4 horses (group 1) had 2 capsules implanted simultaneously; 1 was attached with 1 hemostasis clip (1HEM) and 1 with the helix tacking device (TACK). For additional technique refinement in attempt to achieve 5-7 days of capsule attachment, 5 horses (group 2) then had 1 TACK capsule implanted and 5 horses (group 3) had 1 capsule implanted using 4 hemostasis clips (4HEM).

### pH capsule implantation

Before implantation, gastroscopy was performed using a 3-m endoscope (Olympus Medical Systems Corporation, Tokyo, Japan).

Capsules (Bravo Calibration-free Delivery Device Capsules FGS-0635, Medtronic, Shoreview, MN) then were attached to the glandular mucosa transendoscopically using a 2-m endoscope (Olympus Medical Systems Corporation, Tokyo, Japan), with either hemostasis clips (Resolution 360 ULTRA Clip 2350 mm, Boston Scientific, Marlborough, MA; Figure 2) as previously described^18^^,^^19^ or the helix tacking device (X-Tack Endoscopic HeliX Tacking System 2350 mm, Boston Scientific, Marlborough, MA; Figure 3), according to the group.

**Figure 2:**
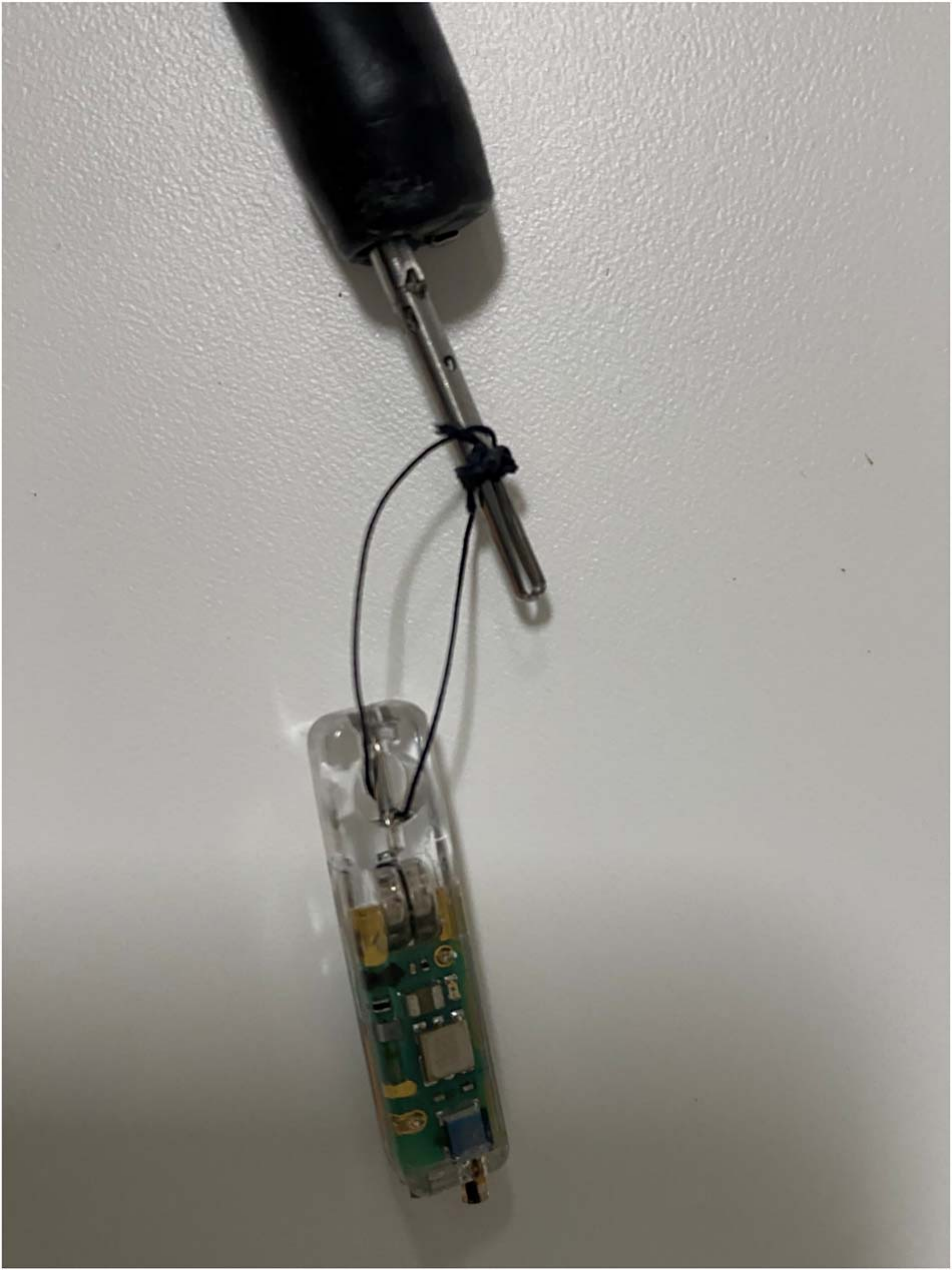
Calibration-free pH capsule attached to the hemostasis clip via a silk suture loop, passed through a 2-m endoscope.

**Figure 3:**
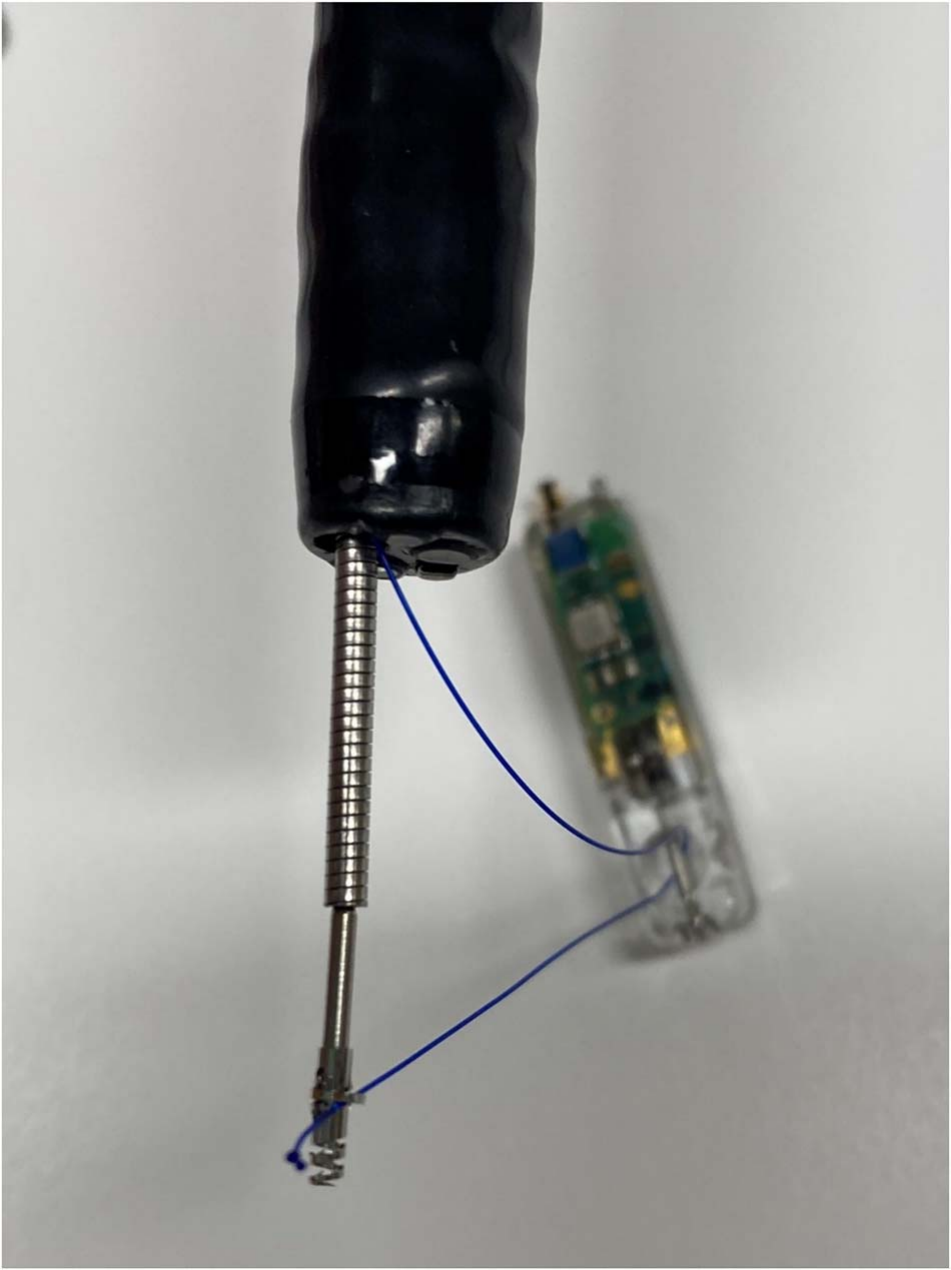
Calibration-free pH capsule attached to the helix tacking device via its preloaded suture, passed through a 2-m endoscope.

### pH recording

Capsules were paired to a wireless recorder (Bravo Calibration-free Reflux Recorder FGS-0634, Medtronic, Shoreview, MN) which was secured to the horse using a surcingle (Televet Electrode Support, Kruuse, Langeskov, Denmark).^18^ Intragastric pH was recorded continuously until the end of the capsule’s battery life or endoscopic confirmation of capsule detachment.^18^^,^^19^

### ERIO administration

After 24 h of baseline pH recording on day 0, ERIO (2 g IM; Omeprazole 100 mg/mL Injectable Suspension BOVA AUS, Caringbah, New South Wales, Australia) was administered on day 1.

### Monitoring

Twice-daily monitoring included physical examination and checking the instantaneous pH for suspected capsule detachment.^18^^,^^19^ Gastroscopy was repeated within 24 h of suspected detachment or loss of signal to confirm attachment status and reassess the mucosa. The safety of the TACK in horses was unknown, and thus gastroscopy was repeated every 14-30 days if tacks remained in situ.

### Data analysis

Data analysis methods are described in the Appendix.

## Results

### pH capsule attachment

All capsules (*n* = 18) were successfully attached to the glandular mucosa and achieved more than 24 h of recording regardless of methodology. Figure 4 shows adjacent capsules attached with 1HEM and TACK, and Figure 5 shows a capsule attached with 4HEM.

**Figure 4:**
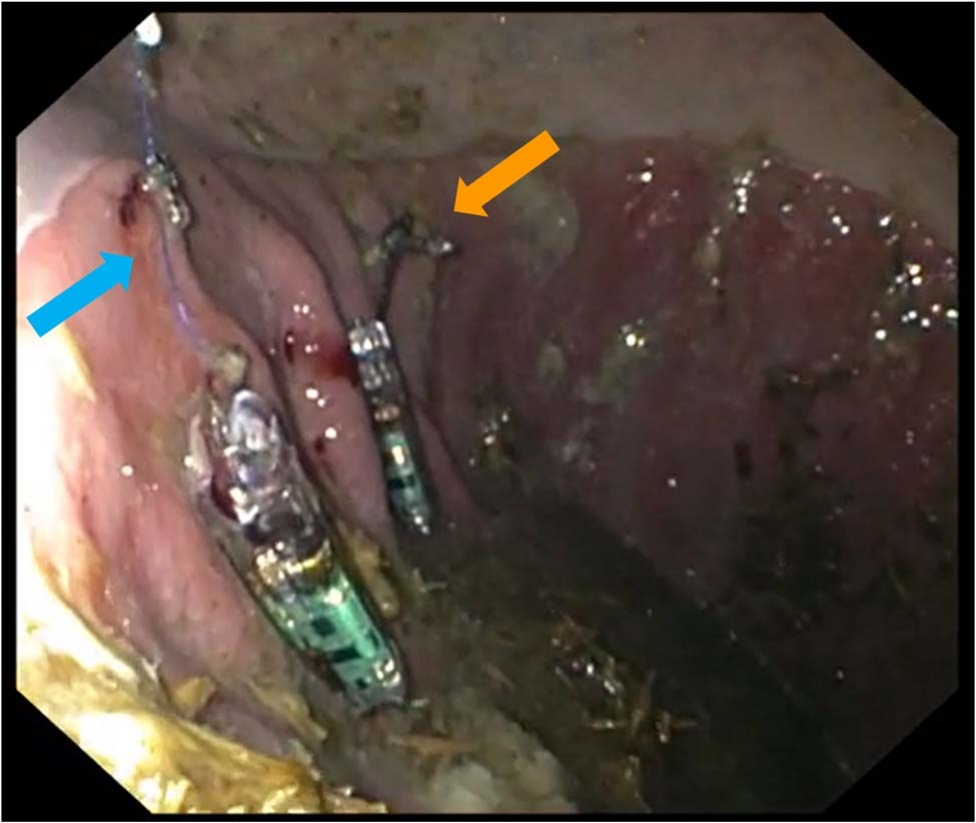
Gastroscopic image showing capsules attached to the glandular mucosa ventral to the margo plicatus with a helix tacking device (TACK; blue arrow) and single hemostasis clip (1HEM; orange arrow). The pH electrode is at the ventral most aspect of the capsule.

**Figure 5:**
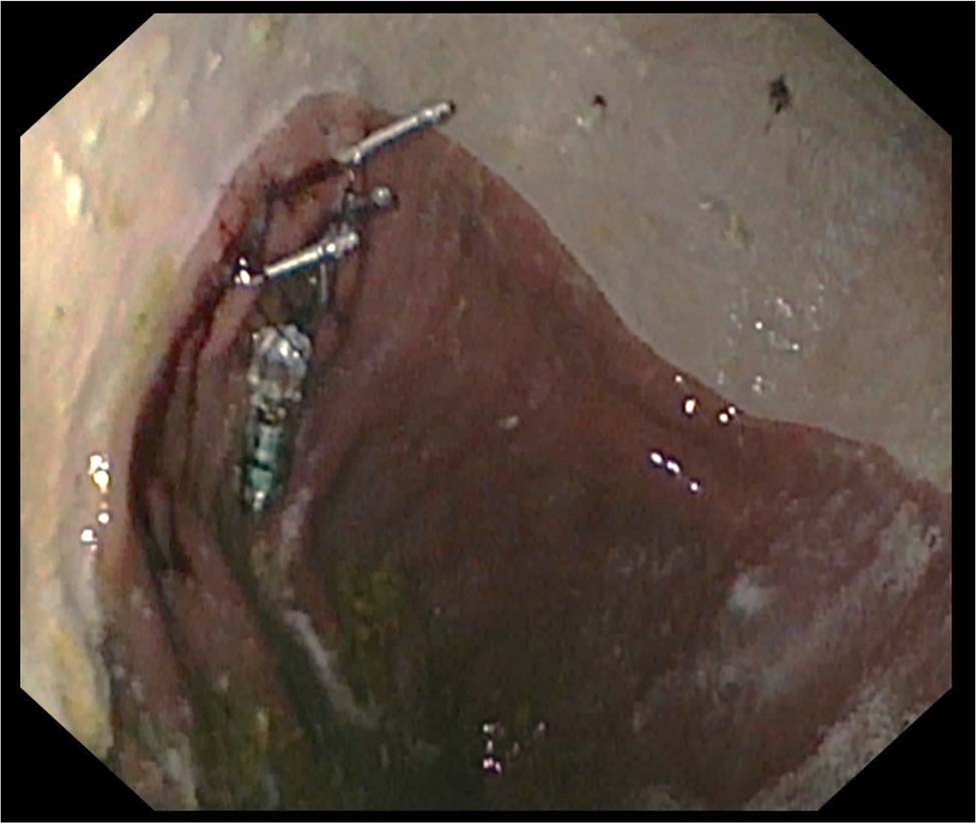
Gastroscopic image showing a capsule attached to the glandular mucosa ventral to the margo plicatus with 4 hemostasis clips (4HEM). The pH electrode is at the ventral most aspect of the capsule.

Capsules remained attached and obtained data for a median (IQR) duration of 51 h (42-65), 129 h (105-256), and 95 h (71-154) for 1HEM, 4HEM, and TACK, respectively (Figure 6). A statistically significant difference in attachment duration was found between methods (*P* = .01) because of a significantly shorter attachment duration for 1HEM than both 4HEM (*P* = .048) and TACK (*P* = .04). No significant difference was found between 4HEM and TACK (*P* = .77). In all 4 horses in group 1 with simultaneously implanted capsules, TACK remained attached longer than 1HEM.

**Figure 6:**
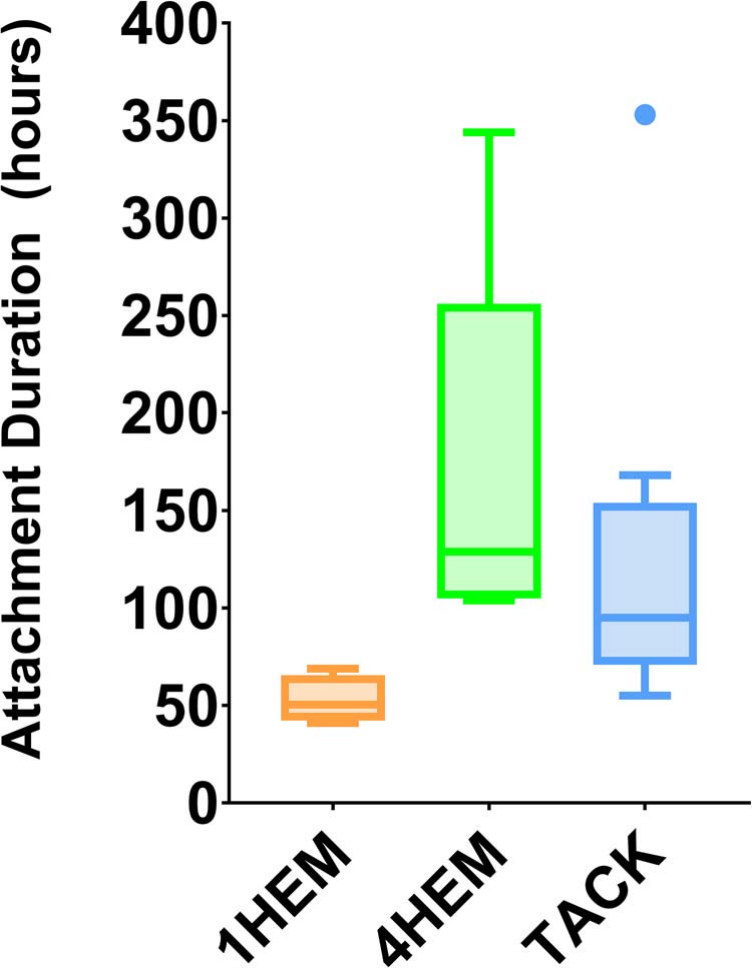
Box and whisker plot showing attachment durations for capsules attached with 1 hemostasis clip (1HEM), 4 hemostasis clips (4HEM), and the helix tacking device (TACK). The median is marked by the horizontal line, the box spans the IQR, and the whiskers span the upper and lower limits. The outliers, calculated by Tukey’s rule, are indicated by circles.

Two capsules (TACK in horse 4 and 4HEM in horse 11) remained attached after the end of the battery life (at 344 and 353 h, respectively) which was confirmed by gastroscopy. Horse 11 was not monitored further, because safety of the hemostasis clips had been demonstrated in previous studies.^18^^,^^19^ Serial monitoring of horse 4 was performed to determine safety of the TACK, and capsule detachment occurred between gastroscopic examinations on days 60 and 90, although the tacks remained in situ 16 months after implantation.

Detachment of the capsules could reliably be identified by a rapid and sustained increase in pH to ≥ 8 and confirmed via gastroscopy in 100% (16/16) of the capsules in which detachment occurred before the end of the battery life. However, in horse 11, a sustained increase in pH ≥ 8 was observed from the end of day 3 to the end of day 11, despite serial gastroscopy confirming the capsule to be in situ. On day 12, pH then returned to baseline levels until recording stopped at the end of the battery life.

### Pharmacodynamics of ERIO

Median pH was significantly higher on day 1 than day 0 (*P* = .003), with a median (IQR) of 5.95 (5.39-6.37) vs 1.70 (1.53-2.79), respectively. The %tpH > 4 was significantly higher on day 1 than day 0 (*P* < .001) with a median (IQR) of 82.0% (72.7-91.2) vs 14.3% (7.1-23.5), respectively. A representative graph of pH over time is shown in Figure 7.

**Figure 7:**
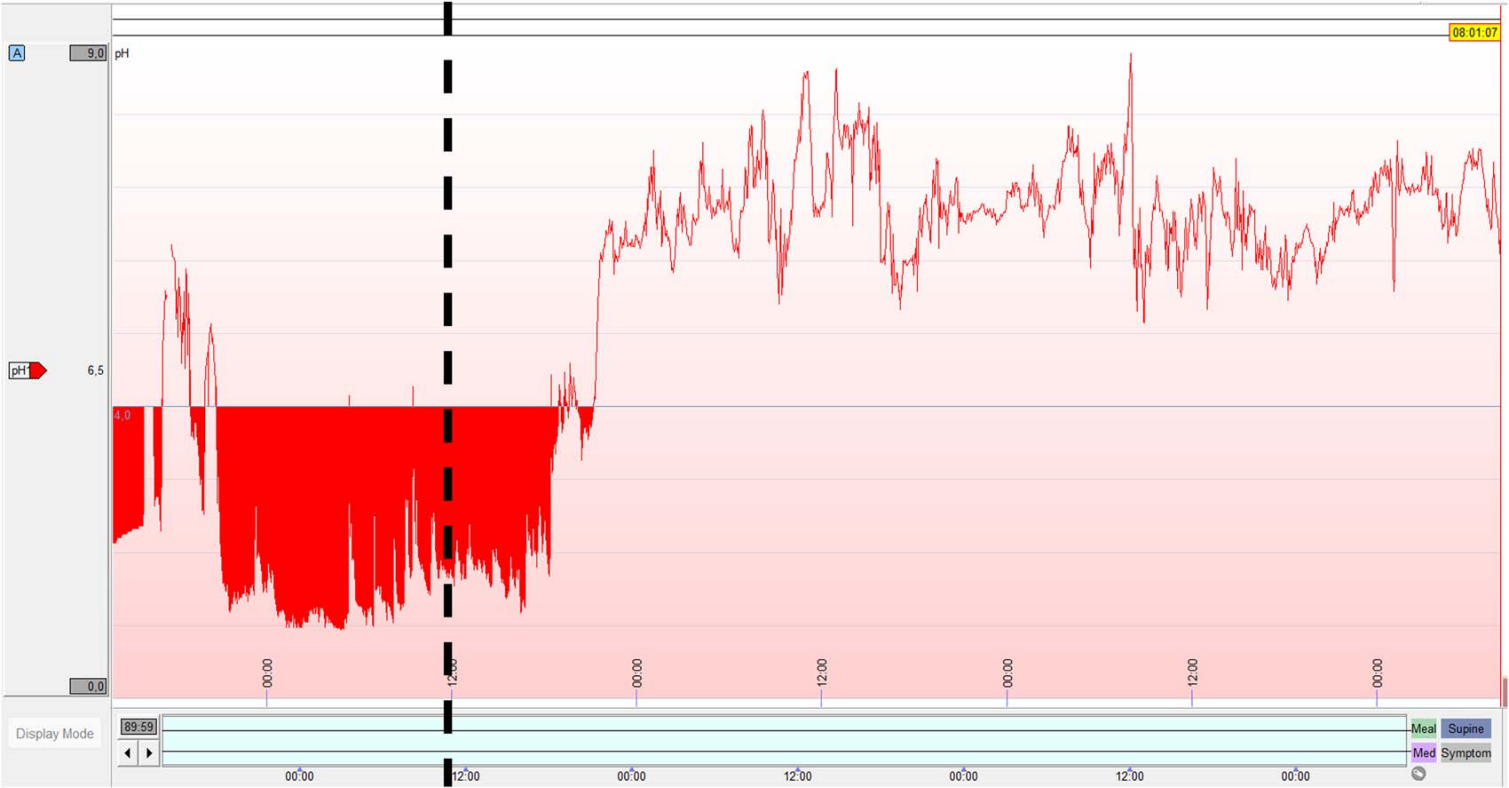
Example of a graph of pH over time generated by the Medtronic Reflux pH software before and after ERIO administration (indicated by the black dashed line). Time (24-h clock) is on the x-axis and pH is on the y-axis. pH < 4 is highlighted by red shading.

Table S1 shows the %tpH > 4 for each day for the total recording duration of each horse (*n* = 12). Substantial individual variation was observed.

A complete pH recording was available for 6/12 horses for days 0-5, representative of the commonly used 5-day dosing interval. Figure 8 shows the median pH and %tpH > 4 over the 6-day period from day 0 through day 5 in this subgroup of horses. Both median pH (*P* < .004) and %tpH > 4 (*P* < .01) were significantly higher than day 0 at each subsequent time point (Tables S2 and S3). The total %tpH > 4 exceeded 66% throughout the 5-day dosing interval in 4/6 horses (Table S1).

**Figure 8:**
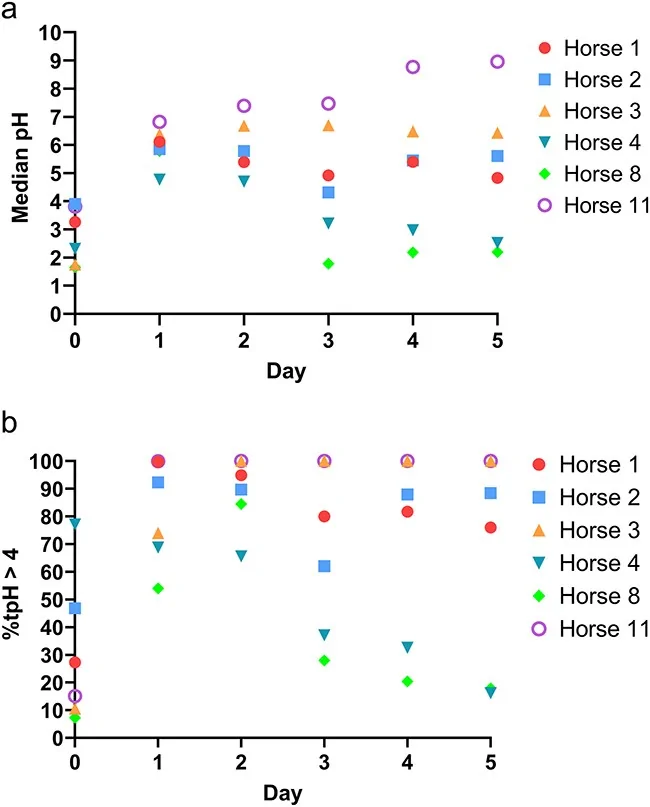
Scatter plots showing the median pH (a) and %tpH > 4 (b) from day 0 through day 5 for horses (*n* = 6) with a complete pH recording throughout the 5-day dosing interval.

### Gastroscopy findings

Initial gastroscopic examination on day 0 identified squamous disease in 8/12 of horses, of which 6/8 were grade 2 and the remaining 2/8 were grade 3. Repeat gastroscopy 3-15 days after ERIO administration identified complete resolution of squamous lesions in 100% of horses (8/8). One horse had a glandular polyp in the pyloric antrum which was unchanged upon repeat gastroscopy. No other glandular lesions were identified in any horse.

Mild transient mucosal hemorrhage was observed during clip placement in 1/4 of 1HEM and 5/5 of 4HEM, as well as during tack placement in 9/9 of TACK. At repeat gastroscopy, focal areas of mild erythema and raised mucosa were identified at the attachment sites for both clips and tacks in all horses (Figure 9).

**Figure 9:**
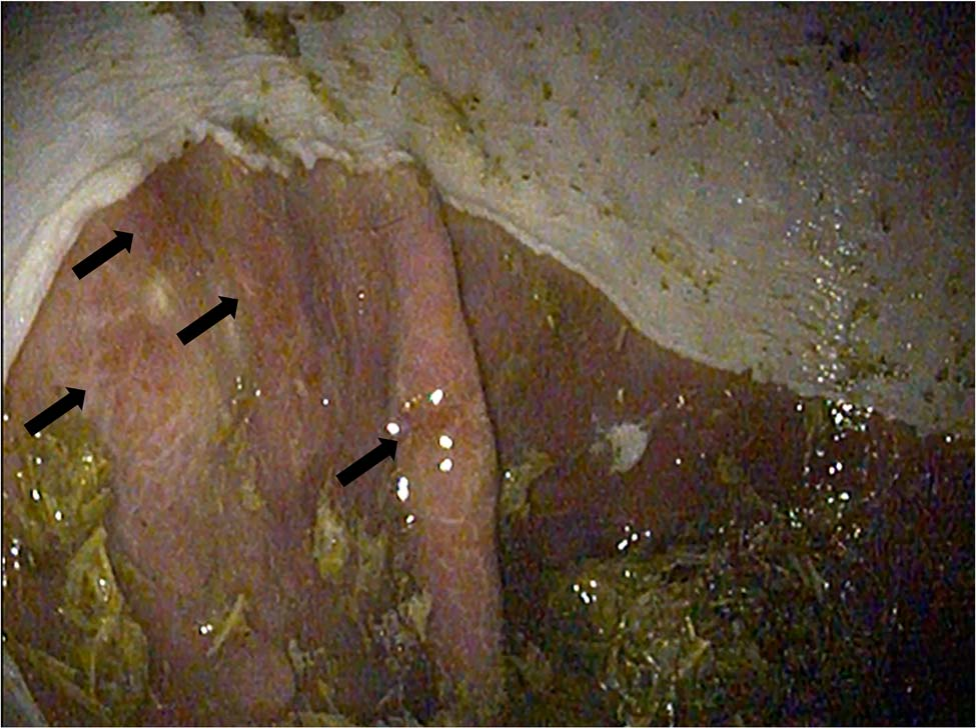
Gastroscopic image showing focal areas of raised erythematous glandular mucosa (black arrows) subsequent to detachment of the hemostasis clip and tacking device.

Repeat gastroscopy confirmed that the TACK capsule of horse 4 and the 4HEM capsule of horse 11 remained in situ after the end of the battery life at 344 and 353 h, respectively. Serial gastroscopic monitoring of the TACK capsule showed the tacking device and capsule in situ on day 30 and day 60. The capsule was no longer present on day 90, but the tacking device was still in situ, although visualization was poor. The horse has since received regular gastroscopic examinations while enrolled in another study and the tacking device was still present 16 months after implantation. Serial evaluation identified a single residual tack implanted in the mucosa with the remaining tacks and cinch held together by the suture, and the surrounding mucosa was healthy with no evidence of inflammation.

### Monitoring

The capsules, hemostasis clips and helix tacking device were well tolerated by all horses with no complications. Elimination of the capsules from the gastrointestinal tract was not confirmed. One horse had a mild, non-painful injection site swelling after ERIO administration which resolved uneventfully within 24 h.

## Discussion

We refined the technique for wireless intragastric pH capsule placement in horses, resulting in improved attachment and recording durations. All 3 minimally invasive methods were safely and readily performed in standing sedated horses and achieved > 24 h of recording, which represents a substantial improvement on the initially described technique.^18^ Our results also demonstrate that the capsules can detect the effect of ERIO on intragastric pH, which was similar to a previous report.^4^ This finding confirms that the technique is a viable model for intragastric pH measurement in pharmacodynamic studies.

In comparison to previously used methods for intragastric pH measurement,^8^^,^^12^^,^^14^ wireless capsule placement is minimally invasive and provides a known fixed electrode location. The transendoscopic technique described in foals with the larger hemostasis clip^19^ was employed in our study, which markedly decreased procedural time and difficulty, and allowed for more precise placement. Using the 2350 mm devices through the 2-m endoscope permitted glandular capsule placement ventral to the margo plicatus in our study population. However, a more ventral location would be desirable, and this length is likely to be insufficient for capsule placement in larger breed horses. The availability of longer hemostasis clips for use through the biopsy channel of a 3-m gastroscope would enable placement in the ventral glandular fundus and pyloric antrum.

When comparing the attachment methods used in our study, 1HEM provided a reliable model for 24 h of recording and is the least expensive and least invasive option if ≤ 24 h of recording is required. Recordings of intragastric pH for 5-7 days were achieved in foals with 1HEM,^19^ and this increased duration compared to adult horses could be attributed to differences in diet, particularly roughage intake. However, longer and more consistent attachment durations were achieved in adult horses in our study than in the original pilot study,^18^ suggesting that the improved retention also may reflect the refined technique and not solely the difference in signalment. Although 5-7 days of capsule attachment was not reliably achieved, attachment with 4HEM appears optimal for more prolonged studies, with consistent attachment durations of at least 4 days. Although long durations were achievable with TACK, substantial variation occurred that did not improve with technique proficiency. No significant difference was found between 4HEM and TACK, but this finding may represent a type II error because of small sample sizes, because attachment duration for 4HEM generally was higher with less variation. The main limitation of our study was the small sample size and unequal subgroups. An iterative process was used to optimize the technique to best achieve the aims of the study. Doing so allowed for refinement and modification of the methodology but resulted in unequal group sizes. Shorter and more variable attachment durations were achieved with TACK than anticipated, which led to fewer complete datasets for the 5- and 7-day ERIO dosing intervals than desired. Based on our results, it is recommended that the 4HEM method be used in future pharmacodynamic studies. Study design also should include the option for capsule replacement in the event of premature detachment, as is described in dogs,^23^ although doing so would require additional periods of feed-withholding and sedation.

The helix tacking device required more skill to operate than did the clips, as is reported in human medicine,^22^ resulting in increased procedural time. Additional technical limitations were encountered during adaptation of the device for horses. It is important that the tack is advanced at a perpendicular angle to the mucosa to achieve deep submucosal and intramuscular fixation. However, the limited length of the device often precluded an optimal angle at the desired glandular location in the stomach of the horse. The implanted portion of the tack is also only 3.5 mm in length. Consequently, tack placement was comparatively more superficial than in humans, which likely increased the risk of dislodgement. A longer device with larger tacks might facilitate tack placement and therefore improve retention in horses, but such a device is not commercially available. Furthermore, although a ``figure-of-8 pattern” of tack placement was used in the current study, the ideal suture tension and patterns are not well established, even for the intended use for defect closure in people.^21^^,^^24^ The observed benefits of the helix tacking device over hemostasis clips in human medicine may not have translated to its use in horses because of anatomical differences, as well as increased forces on the tacks exerted by the capsule and ingested roughage. The device has been successfully used to attach a PEG-jejunal tube^25^ and lumen apposing metal stents (LAMS) in humans,^26^ but fewer vertical pulling forces are expected with these applications. The helix tacking device is substantially more expensive than hemostasis clips, and our results do not justify its use for this purpose. We did however demonstrate safety of the TACK in horses, and its use could be considered for alternative applications in horses such as defect closure. In 1 horse, a tack has remained in situ for over 16 months without incident. We initially planned to attempt removal of any tacks left in situ at 90 days, but because previous attempts to remove tacks in humans have been unsuccessful^28^ and no adverse effects were associated with the tack in situ, it was elected to leave it in place in this horse. Albeit not directly comparable, the device is considered to be very safe in humans^27^ and tack retention has been reported for up to 9 months with no complications, after which no further follow-up was performed.^28^ Based on the observations of our study, continued monitoring beyond the end of the capsule battery life is therefore not considered to be of benefit, and both clips and tacks are expected to slough uneventfully with time.^18^^,^^19^^,^^28^

For most horses in our cohort, a rapid and sustained increase in pH > 8 was consistent with capsule detachment, as reported in other studies.^18^^,^^19^ In horse 11, however, an 11-day period of pH > 8 occurred despite endoscopic confirmation of capsule attachment. The possibility of adherent mucus, saliva or feed material causing electrode interference was excluded based on serial endoscopic evaluation, including thorough rinsing of the capsule with water through the biopsy channel. Because pH values subsequently returned to baseline on day 12, this pH profile is most likely explained by effective and prolonged acid suppression in this horse after the administration of ERIO. Although this magnitude of acid suppression previously has been described with ERIO,^4^ gastric pH had not been measured beyond 7 days after administration. When undertaking pharmacodynamic studies investigating acid suppressive medications, it therefore may be advisable to confirm capsule detachment with gastroscopy or continue pH measurement beyond the expected duration of acid suppression to monitor for a return to baseline. An additional benefit of the capsules is that they are calibration-free, which avoids loss of data caused by pH drift or calibration failure,^9^^,^^29^ which is especially important for prolonged recordings.

A longer duration of recording is particularly useful for interventional or pharmacodynamic studies, where it is desirable to obtain a period of baseline recording followed by continuous recording after drug administration. We demonstrated that the wireless capsules could successfully and reliably detect the effect of ERIO on intragastric pH, with results similar to a previous study that utilized PEG tubes.^4^ In that study, pH was measured at 2 locations on the pH probe; “location 1” measured pH 10-20 mm from the glandular mucosa and is accordingly the most similar to the electrode location on the pH capsule. The 2 electrode locations, however, are not directly comparable, because the PEG tube is positioned more ventrally within the glandular fundus than is currently possible with capsule placement. Overall, however, the findings are similar and suggest that the capsule technique can be used as a suitable pharmacodynamic model.

One notable difference between the 2 studies is the variation in baseline pH observed in our study, emphasizing the importance of obtaining day 0 data before treatment. Although baseline data were recorded for each horse, a control group was not included, which represents a limitation of the study. Interestingly, the baseline %tpH > 4 in 1 horse was actually higher on day 0 than on days 1 and 2. This finding was thought to reflect an abnormal increase in pH on day 0 rather than lack of ERIO efficacy, because pH decreased to more expected levels from day 3 onward. The reason for this initial increase on day 0 is unclear but could represent possible effects of sedation, altered motility, enterogastric reflux, or feeding after fasting for gastroscopy and capsule placement on intragastric pH.^7^^,^^30–33^ Substantial individual variation in the magnitude and duration of acid suppression was evident in both studies,^4^ and is particularly apparent in our population. This observation might be attributed to variability in absorption or elimination of the drug or both and merits further investigation. This variation primarily resulted in waning acid suppression before the end of the dosing interval, which was observed as early as day 3 in our study. Conversely, 1 horse experienced total acid suppression for 11 days after a single dose of ERIO, which has not been reported previously. Another possible explanation, and limitation of the study, is that all horses received a 2 g dose regardless of body weight. This dosing strategy was used in previous ERIO investigations^4^^,^^5^ and reflects the clinical situation because ERIO is supplied as individual single use vials containing 20 mL of 100 mg/mL omeprazole. Moreover, 2 g represents a relative over- rather than under-dose for most horses in the study population based on a 4 mg/kg dosage.^4^

Superior healing rates have been reported with 5- rather than 7-day dosing intervals of ERIO,^5^ particularly for glandular disease, and results from both ERIO pharmacodynamic studies^4^ support this dosing regimen. When 4 doses of ERIO were administered at 5- vs 7-day intervals, healing was achieved in 97% vs 82% of cases for squamous disease and 93% vs 69% for glandular disease.^5^ Optimizing treatment regimens for glandular disease is especially important, because healing rates as low as 23% have been reported with ERIO administered every 7 days.^34^ In our study, a single dose of ERIO resulted in complete healing of squamous disease in 100% of horses. Healing has not been evaluated previously after a single dose, although 100% healing rates have been described after 2^4^ and 4 doses^35^ administered at 7-day intervals. These findings suggest that 4 injections may not always be necessary for treatment of squamous disease, and that healing may occur more rapidly as long as adequate and consistent acid suppression can be achieved.^6^ The glandular healing rates achieved with 5-day intervals also support the potential for a shorter overall treatment duration of 3 weeks.^5^ Conclusions regarding the efficacy of ERIO for glandular disease could not be made from our current study because the only glandular lesion identified was a polyp that remained unchanged upon repeat gastroscopy. The disparity in reported healing rates among different studies^4^^,^^5^^,^^34–37^ is likely reflective of the chosen treatment interval and duration, as well as differences in lesion type and severity, husbandry practices, chosen grading systems and the definitions used for healing. However, individual variation in acid suppression also could play a role. In people with gastroesophageal reflux disease, a direct relationship exists between the duration of intra-day acid suppression and healing rates, which is considered to be more important than the magnitude.^6^ Pharmacodynamic targets have not been established in horses, and the threshold for healing of %tpH > 4 exceeding 66% has been extrapolated from human medicine. In our current study, healing of grade 2/4 ESGD was achieved in 2 horses despite %tpH > 4 below 66%. Pharmacodynamic studies have shown that %tpH > 4 may be as low as 30%-45% after PO omeprazole administration^14^^,^^38^ and although correlation with healing was not directly assessed, the studied formulations are widely considered to be effective for ESGD. Establishing the relationship between intragastric pH and healing of both squamous and glandular disease would be beneficial in future studies to determine whether the target of %tpH > 4 exceeding 66% is an appropriate benchmark in horses.

The magnitude and duration of acid suppression achieved with ERIO in both our current and a previous pharmacodynamic study^4^ are superior to that reported for PO omeprazole.^14^^,^^38^ Improved healing rates have also been described with ERIO compared with PO omeprazole in some^36^^,^^37^ but not all^34^ studies. Extended-release injectable omeprazole provides additional benefits over PO formulations because it circumvents the need for fasting before administration, and the dosing regimen can lead to improved client and patient compliance. Mild injection site reactions occurred in 7.1% of ERIO injections in our study, which is similar to several previous reports,^36^^,^^37^ but other studies reported a lower rate of reactions.^5^ Acute severe systemic reactions have also been reported recently in 2 horses after ERIO administration, which is a complication that had not been recognized previously.^39^ A different ERIO formulation is also available but is associated with lower healing rates and higher numbers of injection site reactions, and its effect on intragastric pH has not been measured.^40^ Both oral and injectable forms of omeprazole have limitations, and novel medications such as potassium-competitive acid blockers may provide an alternative for acid suppression in the horse.^41^

## Conclusion

We refined the technique for minimally invasive wireless measurement of intragastric pH in horses and achieved longer and more consistent attachment durations. The pH capsules could detect and demonstrate a treatment effect of ERIO, providing a feasible model for future pharmacodynamic and interventional studies. Our results support the use of a 5-day dosing interval for ERIO but highlight individual variation, which should be considered, particularly in nonresponsive horses.

## Supplementary Material

Supplementary_materials_aalag287

## Off-label antimicrobial declaration

The authors declare no off-label use of antimicrobials.

## Institutional animal care and use committee or other approval declaration

Approved by the University of Queensland Animal Ethics Committee (2025/AE000419).

## Appendix

### Materials and methods

#### Study cohort and design

Twelve healthy adult mares (8 Standardbreds and 4 Australian Stock Horses) from the University of Queensland Equine Unit, aged 6-27 years and weighing 400-525 kg, were enrolled in a prospective interventional study. Complete physical examinations were performed before enrollment to exclude the presence of clinical disease. Horses were housed in groups of 2-4 in grass paddocks with ad libitum lucerne hay and free access to fresh water. No forced exercise was undertaken during the study period. The University of Queensland Animal Ethics Committee approved the research protocol (2025/AE000419).

An iterative process was used to develop an optimized technique and maximize attachment duration, with the goal of achieving 5-7 days of capsule attachment. To directly compare methods and control for individual horse variability, 4 horses (group 1) initially had 2 capsules implanted simultaneously; 1 was attached with 1 hemostasis clip (1HEM) and the other with the helix tacking device (TACK). There were some initial technical challenges using the TACK, so an additional 5 horses (group 2) had 1 capsule implanted with TACK for technique refinement. Because of persistent variability of attachment duration with TACK despite proficiency with the device, 5 horses (group 3; including 2 horses re-used from group 1) had 1 capsule implanted using 4 hemostasis clips (4HEM) in attempt to more consistently achieve 5-7 days of attachment.

#### Gastroscopy

Before gastroscopy and capsule placement on day 0, feed was withheld for 16 h and water for 2 h. Horses were sedated with 0.02-0.04 mg/kg acepromazine IV (Acepril-10, TROY Laboratories Pty Ltd, Glendenning, NSW, Australia) 30 min before, and 0.01 mg/kg detomidine hydrochloride (Sedator, Randlab Pty Ltd, Revesby, NSW, Australia) and 0.01 mg/kg butorphanol tartrate (Butorphanol, Randlab Pty Ltd, Revesby, NSW, Australia) IV immediately before gastroscopy.

Gastroscopy was performed using a 3-m endoscope (Olympus Medical Systems Corporation, Tokyo, Japan). The presence of squamous or glandular ulceration was assessed and graded^1^; exclusion criteria comprised grade 4 equine squamous gastric disease^42^ or severe equine glandular gastric disease.^1^

#### Preparation of the pH capsules

A wireless, calibration-free pH capsule (6 × 5.5 × 25 mm; Bravo Calibration-free Delivery Device Capsules FGS-0635, Medtronic, Shoreview, MN) was used. The capsule was removed from the manufacturer’s delivery system and attached to the glandular gastric mucosa transendoscopically using a 2-m endoscope (Olympus Medical Systems Corporation, Tokyo, Japan) with either 1 or 4 hemostasis clips (Resolution 360 ULTRA Clip 2350 mm, Boston Scientific, Marlborough, MA) as previously described,^19^ or a helix tacking device (X-Tack Endoscopic HeliX Tacking System 2350 mm, Boston Scientific, Marlborough, MA), according to the group. Capsule preparation and implantation are detailed below. The capsules then were paired to the wireless recorder (Bravo Calibration-free Reflux Recorder FGS-0634, Medtronic, Shoreview, MN).

##### Hemostasis clip preparation

A hemostasis clip was passed through the biopsy channel of the 2-m endoscope. Once through, the capsule was attached to the clip using a 4-cm loop of silk suture (Ethicon 3-0, Johnson & Johnson Medical Pty Ltd, North Ryde, NSW, Australia). First, the suture loop was passed around the clip and looped through itself. Then, the teeth of the hemostasis clip were opened, the loop was wrapped around the teeth of the hemostasis clip in a figure-of-8 pattern, and the teeth were closed. The suture loop then was attached to the capsule; the loop was placed into the capsule suction well and the capsule delivery system was deployed, locking a trocar in place which secured the suture loop in the suction well. This procedure released the capsule from the delivery system, leaving it attached to the hemostasis clip at the end of the endoscope (Figure 2). For the 4HEM capsules, an additional suture loop was also placed into the suction well before deploying and locking the trocar into place to provide additional attachment points for the subsequent hemostasis clips.

##### Helix tacking device preparation

The helix tacking device consists of four 5-mm barbed helix tacks connected to each other via 3-0 polypropylene suture, which runs through an eyelet on each tack. The first tack is preloaded onto the device catheter, with 3 additional reloadable tacks available on the loading card. The tacking device catheter, with the first tack preloaded, was passed through the biopsy channel of the 2-m endoscope. Once through, the capsule was attached to the tacking device using the preloaded polypropylene suture. The length of suture attached to the eyelet of the tack was loosened to create slack and form a loop. The suture loop then was attached to the capsule as described above in “Hemostasis clip preparation.” This procedure released the capsule from the delivery system, leaving it attached to the tacking device at the end of the endoscope (Figure 3).

#### pH capsule placement

A 1800 mm nasogastric overtube (12 mm internal diameter, Dural Irrigation, Dural, New South Wales, Australia) was placed to prevent trauma to the nares, pharynx, or esophagus by the clip or tacking device and capsule. The endoscope with either the clip or tacking device and capsule attached then was advanced into the stomach through the tube. Once through the cardia, the endoscope was advanced beyond the overtube and directed to the glandular mucosa ventral to the margo plicatus at the greater curvature.

##### Hemostasis clip method

The clip-capsule device then was advanced beyond the endoscope, and the hemostasis clip was opened. Mucosal tissue was grasped with the clip, adequate tissue attachment was visually confirmed, and the clip was closed and deployed. For 4HEM capsules, an additional 3 hemostasis clips were passed sequentially down the biopsy channel and used to attach the suture loops to the mucosa at additional points. The endoscope and overtube were removed.

##### Helix tacking method

The tacking device with the capsule attached then was advanced beyond the endoscope, and the tacking device was attached to the mucosa as follows. The first tack was advanced and drilled into the tissue, adequate tissue attachment was visually confirmed, and the tack was deployed. The remaining 3 tacks were sequentially loaded onto the device catheter, fed down the biopsy channel along the preloaded suture, and placed as described for the first tack in a ``figure-of-8 pattern.”^21^ This approach provided a total of 4 points of fixation. The suture cinch (Suture Cinch—Long, Boston Scientific, Marlborough, MA) then was passed down the biopsy channel while tension was applied to the suture. The cinch then was deployed, which locked the tacks in place and cut the excess suture. The endoscope and overtube were removed. In group 1, the TACK capsule was implanted first and then the endoscope was removed and prepared according to the “Hemostasis clip method” for the 1HEM capsule which then was implanted adjacent to the TACK.

For all horses, access to pasture and ad libitum feed and water were reintroduced once sedative effects subsided.

### pH recording

After capsule placement, connection to the recorder was confirmed. The recorder was secured to the horses using a surcingle (Televet Electrode Support, Kruuse, Langeskov, Denmark) and fixed with adhesive bandage (ZebraPlast 7.5 cm Adhesive Elastic Bandage, ZebraVet, Darra, QLD, Australia). The recorder was placed inside a waterproof plastic zip lock bag and then inside the surcingle pouch behind the point of the left elbow to ensure proximity to the capsule and maximal protection.

The recorder collected pH data from the capsules via telemetry every 6 s. Intragastric pH data were recorded continuously until the end of the capsule’s battery life (reported to be 14 days) or endoscopic confirmation of capsule detachment.^18^^,^^19^ Because of the maximum study duration permitted by the recorder, data were uploaded every 96 h, and a new recording started.

### ERIO administration

After 24 h of baseline pH recording on day 0, all horses received ERIO (2 g IM; Omeprazole 100 mg/mL Injectable Suspension BOVA AUS, Caringbah, New South Wales, Australia) on day 1. Extended-release injectable omeprazole was administered into the left or right gluteal muscles after preparation of the injection site with chlorhexidine scrub and isopropyl alcohol.

### Monitoring

Horses were monitored for adverse effects associated with capsule attachment, repeated gastroscopy, sedation or withholding of food. Full physical examination was performed twice daily including before sedation and gastroscopy. Fecal output and food and water consumption were monitored twice daily. The instantaneous pH displayed on the recorder was monitored twice daily for suspected capsule detachment, as indicated by a rapid and sustained increase in pH ≥ 8.^18^^,^^19^

### Follow-up gastroscopy

Gastroscopy was repeated to confirm attachment status within 24 h of suspected detachment or the end of the capsule’s battery life. Feed was withheld for 12 h before. Horses were sedated with 0.1 mg/kg detomidine hydrochloride or 0.3-0.4 mg/kg xylazine IV and gastroscopy then was performed as described in “Gastroscopy.” The mucosa was also examined for any potential consequences of attachment, and development of or change in gastric lesions. If hemostasis clips remained in situ, ongoing monitoring was not performed because safety of the hemostasis clips has been demonstrated in previous studies.^18^^,^^19^ However, because the safety of the TACK in horses was unknown, if any tacks remained in situ gastroscopy was repeated every 14-30 days for 3 months.

### Data analysis

Every 96 h, after confirmation of capsule detachment, or at the end of the capsule’s battery life, pH data were uploaded onto a computer using commercially available software (Reflux Software Version 6.1, Medtronic, Shoreview, MN). Graphs of pH over time were generated by the software and visually analyzed to determine the point of detachment and calculate attachment duration. If capsules remained attached after the end of the battery life, attachment duration was calculated up until the end of the battery life and subsequent loss of signal, because continuous pH recording is required to determine the precise point of detachment. The percentage of time the pH was less than 4 (%tpH < 4) was calculated directly by the software for each day and the percentage of time the pH was above 4 (%tpH > 4) was derived. Raw data were then exported as.txt files and imported into commercially available software (Microsoft Excel v16.0, Microsoft, Redmond, WA). For continuous data, a Shapiro–Wilk test was performed to assess data distribution. The median pH was calculated for each day. Descriptive statistics were reported as median (IQR) for non-normally distributed data and frequencies and percentages for categorical data. A Kruskal–Wallis test was performed to determine whether there were differences in attachment duration between 1HEM, 4HEM, and TACK, and Mann–Whitney *U* tests were performed with Bonferroni correction for post-hoc analysis. The longest pH recording from each horse was extracted for pharmacodynamic analysis of ERIO. To determine whether the capsules could detect a treatment effect of ERIO, Wilcoxon matched-pairs tests were performed to compare median pH and %tpH > 4 between day 0 and day 1. For the subgroup of horses with a complete pH dataset for days 0-5, median pH, and %tpH > 4 for days 1-5 were compared to day 0 using linear mixed modeling for the analysis of repeated measures. Significance was set at *P* < .05.

## Abbreviations

%tpH > 4: percentage of time intragastric pH is above 4
1HEM: 1 hemostasis clip
4HEM: 4 hemostasis clips
EGUS: equine gastric ulcer syndrome
TACK: helix tacking device
ERIO: extended-release injectable omeprazole
LAMS: lumen apposing metal stent
PEG: percutaneous endoscopic gastrostomy

## References

[1] Sykes BW, Hewetson M, Hepburn RJ, Luthersson N, Tamzali Y. European College of Equine Internal Medicine Consensus Statement—equine gastric ulcer syndrome in adult horses. J Vet Intern Med. 2015;29:1288–1299. 10.1111/jvim.13578 PMC4858038 26340142

[2] Argenzio RA . Comparative pathophysiology of nonglandular ulcer disease: a review of experimental studies. Equine Vet J. 1999;31:19–23. 10.1111/j.2042-3306.1999.tb05163.x 10696288

[3] Rendle DI, Gough SL. Pharmaceutical treatment of equine glandular gastric disease: a contextualised review of recent developments. Equine Vet Educ. 2025;37:271–280. 10.1111/eve.14047

[4] Sykes BW, Kathawala K, Song Y, et al. Preliminary investigations into a novel, long-acting, injectable, intramuscular formulation of omeprazole in the horse. Equine Vet J. 2017;49:795–801. 10.1111/evj.12688 28397996

[5] Sundra T, Kelty E, Rendle D. Five- versus seven-day dosing intervals of extended-release injectable omeprazole in the treatment of equine squamous and glandular gastric disease. Equine Vet J. 2024;56:51–58. 10.1111/evj.13938 36977610

[6] Bell NJV, Burget D, Howden CW, Wilkinson J, Hunt RH. Appropriate acid suppression for the management of gastro-oesophageal reflux disease. Digestion. 1992;51 Suppl 1:59–67. 10.1159/000200917 1397746

[7] Murray MJ, Schusser GF. Measurement of 24-h gastric pH using an indwelling pH electrode in horses unfed, fed and treated with ranitidine. Equine Vet J. 1993;25:417–421. 10.1111/j.2042-3306.1993.tb02983.x 8223373

[8] Husted L, Sanchez LC, Olsen SN, et al. Effect of paddock vs. stall housing on 24 hour gastric pH within the proximal and ventral equine stomach. Equine Vet J. 2008;40:337–341. 10.2746/042516408X284673 18267880

[9] Raidal SL, Andrews FM, Nielsen SG, Trope G. Pharmacokinetic and pharmacodynamic effects of two omeprazole formuations on stomach pH and gastric ulcer scores. Equine Vet J. 2017;49:802–809. 10.1111/evj.12691 28432741

[10] Andrews FM . Gastric juice pH measurement in horses: three-quarters of a century of history. Equine Vet Educ. 2015;27:633–636. 10.1111/eve.12505

[11] Toth B, Bertin FR, Miller MA, Charney VA, Kritchevsky JE. Evaluation of a technique for percutaneous endoscopic gastrostomy tube placement in horses. Am J Vet Res. 2014;75:354–360. 10.2460/ajvr.75.4.354 24669920

[12] Sykes BW, McGowan CM, Mills PC. Placement of an indwelling percutaneous gastrotomy (PEG) tube for the measurement of intra-gastric pH in two horses. Equine Vet Educ. 2015;27:627–632. 10.1111/eve.12395

[13] Campbell-Thompson ML, Merritt AM. Effect of ranitidine on gastric acid secretion in young male horses. Am J Vet Res. 1987;48:1511–1515. 10.2460/ajvr.1987.48.10.1511 3674562

[14] Merritt AM, Sanchez LC, Burrow JA, Church M, Ludzia S. Effect of GastroGard and three compounded oral omeprazole preparations on 24 h intragastric pH in gastrically cannulated mature horses. Equine Vet J. 2003;35:691–695. 10.2746/042516403775696339 14649361

[15] Jenkins CC, Blackford JT, Andrews F, et al. Duration of antisecretory effects of oral omeprazole in horses with chronic gastric cannulae. Equine Vet J. 1992;24:89–92. 10.1111/j.2042-3306.1992.tb04795.x

[16] Jenkins CC, Frazier DL, Blackford JT, et al. Pharmacokinetics and antisecretory effects of intravenous omeprazole in horses. Equine Vet J. 1992;24:84–88. 10.1111/j.2042-3306.1992.tb04794.x

[17] Pandolfino JE, Richter JE, Ours T, Guardino JM, Chapman J, Kahrilas PJ. Ambulatory esophageal pH monitoring using a wireless system. Am J Gastroenterol. 2003;98:740–749. 10.1111/j.1572-0241.2003.07398.x 12738450

[18] Hodgson E, Thirouin M, Narayanan P, Romano TR, Wise J, Bond S. A novel placement method of a calibration-free pH capsule for continuous wireless measurement of intragastric pH in horses. J Vet Intern Med. 2025;39:e17273. 10.1111/jvim.17273 PMC11665962 39715411

[19] Hodgson E, Romano T-R, Stewart A, Bond S, Wise J. Prolonged wireless measurement of intragastric pH in foals. J Vet Intern Med. 2026;40:aalaf012. 10.1093/jvimsj/aalaf012 41742481

[20] Canakis A, Dawod SM, Dawod E, et al. Efficacy, feasibility, and safety of the X-Tack Endoscopic HeliX Tacking System: a multicenter experience. J Clin Gastroenterol. 2024;58:1052–1057. 10.1097/MCG.0000000000001977 38277501

[21] Hernandez-Lara A, Garcia G, de Paredes A, Rajan E, et al. Step-by-step instruction: using an endoscopic tack and suture device for gastrointestinal defect closure. VideoGIE. 2021;6:243–245. 10.1016/j.vgie.2021.02.015 PMC8187141 34141961

[22] Hernandez A, Marya NB, Sawas T, et al. Gastrointestinal defect closure using a novel through-the-scope helix tack and suture device compared to endoscopic clips in a survival porcine model (with video). Endosc Int Open. 2021;09:E572–E577. 10.1055/a-1370-9256 PMC8041572 33860074

[23] Odunayo A, Galyon G, Price J, Hecht S, Tolbert MK. Evaluation of a long-acting injectable formulation of omeprazole in healthy dogs. J Vet Intern Med. 2022;36:1416–1421. 10.1111/jvim.16440 PMC9308438 35546514

[24] Canakis A, Al-Sabban A, Irani SS. The endoscopic role and indications of through-the-scope tack and suture system for gastrointestinal closure. Tech Innov Gastrointest Endosc. 2025;27:150898. 10.1016/j.tige.2024.09.005

[25] Rozenberg J, Musallam RJK, Abel WF, Kesar V, Okolo PI, Kesar V. Preemptive fixation of a jejunal enteral tube extension via novel anchoring system. Endoscopy. 2026;58:E107–E108. 10.1055/a-2774-4394 PMC12818999 41558652

[26] Khan HMA, Hussain A, Kumar VCS, Yang D, Hasan MK. Fixation of the proximal flange of a lumen-apposing metal stent using a through-the-scope endoscopic suturing system to prevent stent migration in single-session EUS-directed transgastric ERCP: a pilot study. Gastrointest Endosc. 2024;100:132–135. 10.1016/j.gie.2024.02.013 38382885

[27] Mahmoud T, Wong Kee Song LM, Stavropoulos SN, et al. Initial multicenter experience using a novel endoscopic tack and suture system for challenging GI defect closure and stent fixation (with video). Gastrointest Endosc. 2022;95:373–382. 10.1016/j.gie.2021.10.018 34695421

[28] Mohapatra S, Fukami N. Follow-up outcomes of mucosal defect closures after endoscopic resection using a helix tacking system and endoclips. VideoGIE. 2022;7:268–272. 10.1016/j.vgie.2022.03.002 PMC9263876 35815167

[29] Aristizabal F, Nieto J, Yamout S, Snyder J. Effect of a hay grid feeder on feed consumption and measurement of the gastric pH using an intragastric electrode device in horses: a preliminary report. Equine Vet J. 2014;46:484–487. 10.1111/evj.12175 23991941

[30] Husted L, Sanchez LC, Baptiste KE, et al. Effect of a feed/fast protocol on pH in the proximal equine stomach. Equine Vet J. 2009;41:658–662. 10.2746/042516409X416431 19927584

[31] Doherty TJ, Andrews FM, Provenza MK, Frazier DL. The effect of sedation on gastric emptying of a liquid marker in ponies. Vet Surg. 1999;28:375–379. 10.1111/j.1532-950X.1999.00375.x 10493642

[32] Sutton DGM, Preston T, Christley RM, et al. The effects of xylazine, detomidine, acepromazine and butorphanol on equine solid phase gastric emptying rate. Equine Vet J. 2002;34:486–492. 10.2746/042516402776117818 12358052

[33] Merritt AM . Normal equine gastroduodenal secretion and motility. Equine Vet J. 1999;31:7–13. 10.1111/j.2042-3306.1999.tb05161.x 10696286

[34] Pratt SL, Bowen M, Hallowell GH, Shipman E, Bailey J, Redpath A. Does lesion type or severity predict outcome of therapy for horses with equine glandular gastric disease?—a retrospective study. Vet Med Sci. 2023;9:150–157. 10.1002/vms3.1034 PMC9857143 36495212

[35] Rendle D, Gosling L, Platt A, Duff A. Efficacy of long-acting injectable omeprazole (LAIOMEP) in the management of equine glandular gastric disease (EGGD) and equine squamous gastric disease (ESGD). Equine Vet J. 2018;50:14. 10.1111/evj.20_13008

[36] Gough S, Hallowell G, Rendle D. A study investigating the treatment of equine squamous gastric disease with long-acting injectable or oral omeprazole. Vet Med Sci. 2020;6:235–241. 10.1002/vms3.220 PMC7196684 31945806

[37] Gough S, Hallowell G, Rendle D. Evaluation of the treatment of equine glandular gastric disease with either long-acting-injectable or oral omeprazole. Vete Med Sci. 2022;8:561–567. 10.1002/vms3.728 PMC8959258 35167731

[38] Sykes BW, Underwood C, Greer R, McGowan CM, Mills PC. The effects of dose and diet on the pharmacodynamics of omeprazole in the horse. Equine Vet J. 2017;49:525–531. 10.1111/evj.12630 27554924

[39] Vandecandelaere M, Sykes BW, Sullivan S, Thiris O, Ware S, van Galen G. Acute and severe reactions after administration of a compounded extended-release injectable omeprazole in two horses. Equine Vet Educ. 2026;24:573–580. 10.1111/eve.70046

[40] Lehman ML, Bass L, Gustafson DL, Rao S, O’Fallon ES. Clinical efficacy, safety and pharmacokinetics of a novel long-acting intramuscular omeprazole in performance horses with gastric ulcers. Equine Vet Educ. 2022;34:573–580. 10.1111/eve.13546

[41] Morales CJ, Sykes BW, McKemie DS, Kass PH, Knych HK. Vonoprazan pharmacokinetics and effects on gastric pH following administration to fed and fasted horses. Equine Vet J. 2026;58:1363–1371. 10.1111/evj.70128 PMC13447891 41532453

[42] Equine Gastric Ulcer Council . Recommendations for the diagnosis and treatment of equine gastric ulcer syndrome (EGUS): the Equine Gastric Ulcer Council. Equine Vet Educ. 1999;11:262–272. 10.1111/j.2042-3292.1999.tb00961.x

