# Supplementary material for: Prolonged wireless measurement of intragastric pH and pharmacodynamics of extended-release injectable omeprazole in horses
Source: J Vet Intern Med. 2026 Nov 12;40(6):aalag287. doi: 10.1093/jvimsj/aalag287 (PMC13755356; doi:10.1093/jvimsj/aalag287)
Supplement: Supplementary file 1 [file supplementary_materials_aalag287.zip › sub tab 1 cleaned to help author so it is not published with track changes.docx]

**Supplementary Table 1:** Percentage of time the pH was above 4 (%tpH > 4) for each day of recording for each horse (n = 12). Data were excluded in the case of capsule detachment or incomplete 24-hour recordings. Extended-release injectable omeprazole (ERIO) was administered on Day 1. %tpH > 4 values above 66% are indicated in bold.

|  | %tpH >4 | | | | | | | | | | | | | |
| --- | --- | --- | --- | --- | --- | --- | --- | --- | --- | --- | --- | --- | --- | --- |
|  | Day 0 | Day 1 | Day 2 | Day 3 | Day 4 | Day 5 | Day 6 | Day 7 | Day 8 | Day 9 | Day 10 | Day 11 | Day 12 | Day 13 |
| Horse 1 | 27.3 | **99.7** | **94.9** | **80.0** | **81.7** | **76.0** | **79.4** |  |  |  |  |  |  |  |
| Horse 2 | 56.8 | **92.3** | **89.7** | 62.0 | **87.9** | **88.4** |  |  |  |  |  |  |  |  |
| Horse 3 | 10.6 | **74.0** | **100.0** | **100.0** | **100.0** | **100.0** |  |  |  |  |  |  |  |  |
| Horse 4 | 77.1 | **68.8** | 65.6 | 37.1 | 32.6 | 16.2 | 3.9 | 1.6 | 3.0 | 2.6 | 0.5 | 1.9 | 8.9 | 13.4 |
| Horse 5 | 16.8 | **80.8** | **86.0** | **81.8** |  |  |  |  |  |  |  |  |  |  |
| Horse 6 | 6.3 | **83.2** | **99.5** |  |  |  |  |  |  |  |  |  |  |  |
| Horse 7 | 22.2 | **90.8** | **100.0** |  |  |  |  |  |  |  |  |  |  |  |
| Horse 8 | 7.3 | 54.0 | **84.5** | 28.0 | 20.4 | 17.9 | 15.4 |  |  |  |  |  |  |  |
| Horse 9 | 0.2 | **87.9** | **95.9** | **70.2** |  |  |  |  |  |  |  |  |  |  |
| Horse 10 | 13.4 | **78.1** | **96.8** | **77.4** |  |  |  |  |  |  |  |  |  |  |
| Horse 11 | 15.1 | **99.9** | **100.0** | **100.0** | **100.0** | **100.0** | **100.0** | **100.0** | **100.0** | **100.0** | **100.0** | **100.0** | 17.9 | 5.3 |
| Horse 12 | 6.2 | 52.4 | **100.0** | **98.1** | 3.0 |  |  |  |  |  |  |  |  |  |
