# Supplementary material for: Prolonged wireless measurement of intragastric pH and pharmacodynamics of extended-release injectable omeprazole in horses
Source: J Vet Intern Med. 2026 Nov 12;40(6):aalag287. doi: 10.1093/jvimsj/aalag287 (PMC13755356; doi:10.1093/jvimsj/aalag287)
Supplement: Supplementary file 1 [file supplementary_materials_aalag287.zip › sub tab 2 3 cleaned to help author so it is not published with track changes.docx]

**Supplementary Table 2:** Linear mixed model comparing the median pH on days 1-5 to day 0. *P*-values < 0.05 are indicated in bold.

| Median pH | | | | | |
| --- | --- | --- | --- | --- | --- |
| Day | Median | Interquartile Range | Beta (β) | 95% Confidence Interval | *P*-value |
| 0 | 2.79 | 1.75 – 3.81 | **-** | **-** |  |
| 1 | 5.98 | 5.78 – 6.38 | 3.2 | 1.9, 4.4 | **< 0.001** |
| 2 | 5.76 | 5.39 – 6.68 | 3.2 | 1.9, 4.4 | **< 0.001** |
| 3 | 4.62 | 3.21 – 6.70 | 2.0 | 0.7, 3.2 | **0.004** |
| 4 | 5.43 | 2.97 – 6.48 | 2.4 | 1.2, 3.7 | **< 0.001** |
| 5 | 5.22 | 2.52 – 6.43 | 2.3 | 1.0, 3.6 | **< 0.001** |

**Supplementary Table 3:** Linear mixed model comparing the percentage of time the pH is greater than 4 (%tpH > 4) on days 1-5 to day 0. *P*-values < 0.05 are indicated in bold.

| Percentage of time the pH is greater than 4 (%tpH > 4) | | | | | |
| --- | --- | --- | --- | --- | --- |
| Day | Median | Interquartile range | Beta (β) | 95% Confidence Interval | *P*-value |
| 0 | 21 | 11 – 47 | **-** | **-** |  |
| 1 | 83 | 69 – 100 | 51 | 24, 77 | **< 0.001** |
| 2 | 92 | 85 – 100 | 58 | 32, 85 | **< 0.001** |
| 3 | 71 | 37 – 100 | 37 | 11, 64 | **0.008** |
| 4 | 85 | 33 – 100 | 40 | 13, 66 | **0.005** |
| 5 | 82 | 18 – 100 | 36 | 9, 62 | **0.010** |
